# Correction: The impact of tackle football injuries on the American healthcare system with a neurological focus

**DOI:** 10.1371/journal.pone.0201273

**Published:** 2018-07-19

**Authors:** Michael J. McGinity, Ramesh Grandhi, Joel E. Michalek, Jesse S. Rodriguez, Aron M. Trevino, Ashley C. McGinity, Ali Seifi

## Notice of Republication

The original Supporting Information file was published in error. The file was removed in the HTML and PDF versions of this article on July 6, 2018. The article was republished again on July 13, 2018 to update the Data Availability statement for accuracy. Please download this article again to view the correct version. The republished article from July 6, 2018 and the current, republished, corrected article are provided here for reference.

## Supporting information

S1 File Republished version from July 6, 2018. (PDF)

S2 File Republished, corrected article. (PDF)
