# Supplementary material for: Correction: The impact of tackle football injuries on the American healthcare system with a neurological focus
Source: PLoS One. 2018 Jul 19;13(7):e0201273. doi: 10.1371/journal.pone.0201273 (PMC6053222; doi:10.1371/journal.pone.0201273)
Supplement: S1 File — (PDF) [file pone.0201273.s001.pdf]

PLOS ONE

RESEARCH ARTICLE

The impact of tackle football injuries on the American healthcare system with a neurological focus

Michael J. McGinity[1], Ramesh Grandhi[1], Joel E. Michalek[2], Jesse S. Rodriguez[1], Aron M. Trevino[2], Ashley C. McGinity[3], Ali Seifi[1]*

1 Department of Neurosurgery, University of Texas Health Science Center at San Antonio, San Antonio, Texas, United States of America, **2** Department of Epidemiology and Biostatistics, University of Texas Health Science Center at San Antonio, San Antonio, Texas, United States of America, **3** Department of Surgery, University of Texas Health Science Center at San Antonio, San Antonio, Texas, United States of America

* Seifi@uthscsa.edu

Check for updates

OPEN ACCESS

Citation: McGinity MJ, Grandhi R, Michalek JE, Rodriguez JS, Trevino AM, McGinity AC, et al. (2018) The impact of tackle football injuries on the American healthcare system with a neurological focus. PLoS ONE 13(5): e0195827. https://doi.org/10.1371/journal.pone.0195827

Editor: Faramarz Dehghani, Martin Luther University, GERMANY

Received: September 15, 2017

Accepted: March 23, 2018

Published: May 7, 2018

Copyright: © 2018 McGinity et al. This is an open access article distributed under the terms of the Creative Commons Attribution License, which permits unrestricted use, distribution, and reproduction in any medium, provided the original author and source are credited.

Data Availability Statement: All relevant data are within the paper and its Supporting Information files.

Funding: The authors received no specific funding for this work.

Competing interests: The authors have declared that no competing interests exist.

Abstract

Background

Recent interest in the study of concussion and other neurological injuries has heightened awareness of the medical implications of American tackle football injuries amongst the public.

Objective

Using the National Emergency Department Sample (NEDS) and the National Inpatient Sample (NIS), the largest publicly available all-payer emergency department and inpatient healthcare databases in the United States, we sought to describe the impact of tackle football injuries on the American healthcare system by delineating injuries, specifically neurological in nature, suffered as a consequence of tackle football between 2010 and 2013.

Methods

The NEDS and NIS databases were queried to collect data on all patients presented to the emergency department (ED) and/or were admitted to hospitals with an ICD code for injuries related to American tackle football between the years 2010 and 2013. Subsequently those with football-related neurological injuries were abstracted using ICD codes for concussion, skull/face injury, intracranial injury, spine injury, and spinal cord injury (SCI). Patient demographics, length of hospital stay (LOS), cost and charge data, neurosurgical interventions, hospital type, and disposition were collected and analyzed.

Results

A total of 819,000 patients presented to EDs for evaluation of injuries secondary to American tackle football between 2010 and 2013, with 1.13% having injuries requiring inpatient admission (average length of stay 2.4 days). 80.4% of the ED visits were from the pediatric population. Of note, a statistically significant increase in the number of pediatric concussions

over time was demonstrated (OR = 1.1, 95% CI 1.1 to 1.2). Patients were more likely to be admitted to trauma centers, teaching hospitals, the south or west regions, or with private insurance. There were 471 spinal cord injuries and 1,908 total spine injuries. Ten patients died during the study time period. The combined ED and inpatient charges were $1.35 billion.

Conclusion

Injuries related to tackle football are a frequent cause of emergency room visits, specifically in the pediatric population, but severe acute trauma requiring inpatient admission or operative interventions are rare. Continued investigation in the long-term health impact of football related concussion and other repetitive lower impact trauma is warranted.

Introduction

According to the results of the most recent survey by The Harris Poll®, professional football has remained America's favorite sport for the past thirty years [1]. Owing to a heightened awareness of mild traumatic brain injury (TBI) sustained during tackle football, there has recently been increased interest in understanding the medical consequences of football, specifically relating to neurologic injuries. Concussion and spinal cord injury (SCI) have garnered significant attention in illustrating the potential dangers associated with playing football [2]. In addition, researchers are currently investigating a possible connection between repetitive head trauma from American football and chronic traumatic encephalopathy (CTE). Football players have been shown to be twice as likely to sustain a severe injury (i.e., any injury that resulted in a loss of > 21 days of sports participation), than those playing the next most dangerous sport, wrestling [3]. While efforts have been made in implementing injury prevention measures, as well as incorporating new rules and safety regulations (e.g., coach/player education, banning helmet-to-helmet tackles, and institution of return to play guidelines), there is still concern of the violent nature of the sport. A survey through the Physical Activity Council[13] indicated a recent decline in youth football participation, with these frequently discussed risks potentially representing a key factor in parents discouraging their children to play.

The risk of injury associated with playing American football is significant and this risk increases as the competition advances [4]. Past studies have reported all-cause injury rates as high as 8 injuries per 1000 in high school football, 36 per 1000 in college football, and 65 per 1000 in the National Football League (NFL) [5–7]. Furthermore, football has one of the highest incidences of TBI of all major sports, and concussions have been identified as one of the most frequently occurring injuries across all ages [6,8,9]. With recent evidence demonstrating an association between repetitive head trauma and long-term neuropsychiatric and cognitive outcomes, concerns regarding the impact of repeated head injuries are especially noteworthy given that the majority of organized football participants are of the pediatric age [10,11].

The National Emergency Department Sample (NEDS) and the National Inpatient Sample (NIS) represent the largest publicly available all-payer emergency department (ED) and inpatient healthcare databases, respectively, yielding national estimates on ED visits and inpatient stays. NEDS and NIS are components of a set of databases and software tools developed for the Healthcare Cost and Utilization Project (HCUP) [12,13]. We aimed to assess the demographics of patients injured and specific neurological injuries sustained by patients secondary to

tackle football who required ED visits and/or inpatient admissions. The secondary aim was to determine the likelihood of hospital admission based on patient age, type of neurological injury and hospital demographics.

Methods

Data were abstracted from the NEDS and NIS databases from 2010 through 2013. Both databases are included in the HCUP. Unweighted, the NEDS contains data from more than 30 million ED visits each year and the NIS contains data from more than 7 million hospitalizations each year; weighted, each database estimates roughly 135 million ED visits and 35 million hospitalizations each year, comprising a 20% random sample of all ED visits and admissions to community hospitals in the United States [12,13].

The ICD9-CM code E007.0 was used to identify patients with an ED presentation and/or inpatient admission for injury related to American tackle football. Of patients presenting to the ED with code E007.0 in the NEDS file, those with NEDS disposition equal to 9, 'Admitted as inpatient to hospital' were selected as having been admitted to hospital. Over the course of the 4 years of collected data, a total of 118,045,438 charts were queried from the NEDS database with a weighted sum of 529,287,163 charts. Similarly, 29,987,376 charts from the NIS database were queried with a weighted sum of 146,031,246 charts. We report weighted totals using year and hospital-specific weights included with each database with statistical methods for survey designs including the SAS surveyfreq and surveymeans procedures. The significance of associations between categorical outcomes, such as injury type, and admission to hospital (Admitted, Not Admitted) were assessed with weighted mixed effects generalize estimating equations models with adjustment for clustering within hospital using the SAS genmod procedure (SAS Version 9.4 for Windows, SAS Institute, Cary, North Carolina). Similar statistical models were used for continuously distributed outcomes, such as age, total charges, cost, and length of stay. Graphics were made with R. Data were extracted based on ICD-9 and Clinical Classification Software (CCS) diagnostic and procedure codes, shown here in character format. Diagnostic codes for intracranial injury (CCS 233), concussion (ICD-9 8500, 85011, 85012, 8502, 8503, 8504, 8505, 8509), spinal cord injury (CCS 227), all spine injuries (ICD-9 8050.0–805.08, 805.10–805.18, 805.2–805.9, 806.00–806.39, 806.4, 806.5, 806.60–806.62, 806.69–806.72, 806.79, 806.8, 806.9, 349.39, 952.00–952.19, 952.2–95.24, 952.8, 952.9), and skull/face injuries (CCS 228) were abstracted to determine specific neurologic injuries. Patient demographic information, length of hospital stay, cost and charge data, hospital location and designation (trauma center, non-trauma center, teaching facility, non-teaching facility), disposition, and payer type were abstracted. In addition, all patients admitted to an inpatient facility were queried for neurosurgical procedures, including all relevant cranial surgery (ICD-9 procedure codes 01.31, 01.21, 01.24, 01.25, 01.09, 01.26, 01.28, 02.02, 02.99, 01.10, 02.21, 01.39, 01.59) and spine surgery (810.0, 810.1, 810.2, 810.3, 810.4, 810.5, 810.6, 810.7, 810.8, 845.1, 816.2–4, 80.51, 03.09). Patients with at least one occurrence of these codes or groups of codes, as indicated, were identified and categorized with SAS code to permit tabulation and statistical analysis. Throughout, we used 'patient' in reference to individual records in the NIS and NEDS files even though patients have been de-identified.

Results

Over the four-year period from 2010 to 2013, 529 million visits were evaluated in community hospital emergency departments in the United States, and of these, 819,000 with American tackle football-related injuries were seen (0.15%), increasing from 193,826 in 2010 to 213,607 in 2013. Over this time period 9,216 out of all 819,000 ED patients with football injury (1.13%)

were admitted to the hospital where there was an average length of stay of 2.4 days. A total of 8,348 patients in the NIS files for the years 2010, 2011, 2012 and 2013 were coded as E007.0. Thus 868 = 9,216–8348 were coded as E007.0 in the NEDS files with disposition 'Admitted as inpatient to hospital' but were not in the NIS files, preventing us from determining their disposition.

Among patients who presented to the ED with football injuries, patients were more likely to be admitted to the hospital in which the ED was associated if the hospital was designated a trauma center (1.6% vs 0.5%, OR = 3, 95% CI 2.4 to 3.8). Similarly, patients presenting to teaching hospital EDs were more likely to be admitted to that hospital (1.9% vs 0.7%, OR = 2.9, 95% CI 2.2 to 3.7). Patients were also more likely to be admitted to the hospital if they had private insurance compared to Medicaid or self-pay (1.3%%, 0.9%, 0.9%%, respectively). Interestingly, the region of the country was found to be a factor in determining likelihood of admission. Hospitals in the Southern and Western U.S. were more likely to admit patients with football related injuries than those in the Northeast or Midwest. (South 1.25%, West 1.36%, Northeast 0.85%, Midwest 0.85%). Table 1 describes the demographics of patients seen at the ED with football injuries.

The combined inpatient and ED charges over 4 years were 1.35 billion dollars for an average of $337,500,000/year while inpatient cost totaled $102,648,960.

80.4% of all ED visits were patients aged 17 or younger. Of patients presenting to the ED with tackle football injury and diagnosis of concussion, 62,088 of 68,830 (90%) were from the pediatric population (age 17 and younger), which demonstrated a statistically significant increase across time (OR = 1.1, 95% CI 1.1 to 1.2, Fig 1). Diagnostic codes for spinal cord injury (SCI) and all spine injuries revealed 472 and 1908 evaluations, respectively, over the same time frame; however, there was no increase in the number of ED evaluations for these pathologies over this time period.

Among people who presented to the ED with a football injury (Table 2), SCI diagnosis resulted in the highest likelihood of inpatient admission (SCI 43.3%, Other diagnoses 1.1%, OR = 68.5, 95% CI 44.8 to 104.8). A total of 1,908 patients were evaluated for spinal injuries and 472 for spinal cord injuries during the study period.

Table 1. Demographics of patients seen at the ED with football injuries by admission to hospital 2010 to 2013 ((N = 819,000).

Characteristic		Admitted to Hospital		OR (95% CI)
		Yes (N = 9,217)	Total (N = 819,000)	
Male		9062 (1.2)	782589 (95.6)	2.7 (1.9, 3.9)
Age	[0, 17]	2197 (1.4)	160622 (19.6)	1.3 (1.1, 1.5)
	17+	7019 (1.1)	658377 (80.4)	
Payer	Medicare	109 (1.6)	6666 (0.8)	1.2 (0.7, 2.1)
	Medicaid	2532 (0.9)	281859 (34.4)	0.7 (0.6, 0.8)
	Uninsured	1150.4 (0.9)	121678 (14.9)	0.7 (0.6, 0.8)
	Private Insurance	5420 (1.3)	406880 (49.7)	
Region	Northeast	805 (0.8)	95048 (11.6)	0.6 (0.4, 0.9)
	Midwest	1887 (0.9)	222008 (27.1)	0.6 (0.5, 0.8)
	South	3809 (1.3)	302708 (37)	0.9 (0.7, 1.2)
	West	2713 (1.4)	199234 (24.3)	
Hospital	Teaching	5682 (1.9)	296462 (36.2)	2.9 (2.2, 3.7)
	Non-Teaching	3534 (0.7)	522537 (63.8)	
Trauma Status	Trauma	7365 (1.6)	467475 (57.1)	3 (2.4, 3.8)
	Non-Trauma	1851 (0.5)	351524 (42.9)	

https://doi.org/10.1371/journal.pone.0195827.t001

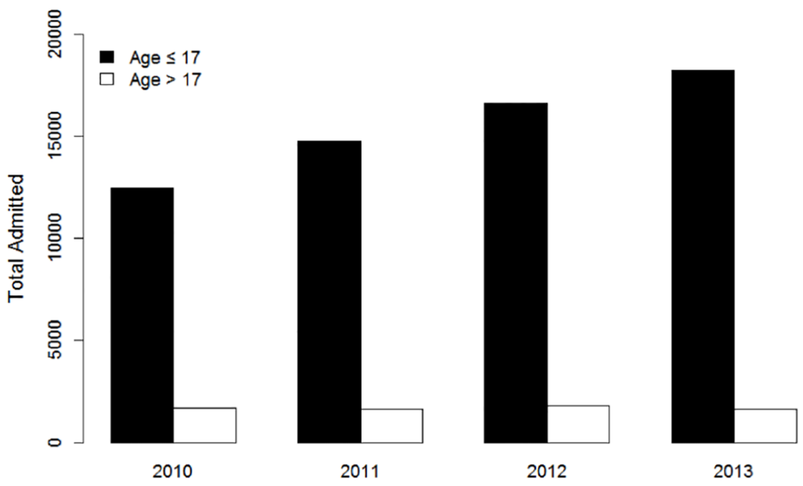

Fig 1. The number of patients admitted to an emergency department with a concussion diagnosis and football injury by age bracket (≤17, >17) from 2010–2013. Among persons ≤ 17 years of age, the number of such admissions increased significantly from 2010 to 2013 (p<0.001).

https://doi.org/10.1371/journal.pone.0195827.g001

Table 2. Specific injuries in patients seen in the ED with football injuries by hospital admission (N = 819,000).

			Admitted to Hospital		OR (95% CI)
**Injury**	**Age**	**Injury Yes/No**	**Yes (N = 9,217)**	**Total (N = 819,000)**	
Concussion	[0,17]	Yes	766 (1.2)	62087	1.2 (1, 1.4)
		No	6253 (1)	596288	
	17+	Yes	133 (2)	6741	1.5 (1, 2.3)
		No	2063 (1.3)	153880	
	All	Yes	900 (1.3)	68830	1.2 (1, 1.4)
		No	8316 (1.1)	750169	
Spinal Cord	[0,17]	Yes	158 (38.6)	410	59.6 (36.8, 96.6)
		No	6860 (1)	657964	
	17+	Yes	46 (74.4)	61	214 (64.7, 707.9)
		No	2150 (1.3)	160559	
	All	Yes	204 (43.3)	472	68.5 (44.8, 104.8)
		No	9011 (1.1)	818525	
All Spine	[0,17]	Yes	335 (21.4)	1569	26.5 (18.9, 37)
		No	6683 (1)	656805	
	17+	Yes	121 (35.9)	337	42.7 (24.6, 74)
		No	2075 (1.3)	160284	
	All	Yes	457 (24)	1908	29.1 (21.6, 39.2)
		No	8759 (1.1)	817090	
Skull and Face	[0,17]	Yes	104 (3)	3444	2.9 (1.8, 4.7)
		No	6915 (1.1)	654931	
	17+	Yes	135 (4.2)	3212	3.3 (2.2, 5)
		No	2061 (1.3)	157409	
	All	Yes	239 (3.6)	6657	3.3 (2.5, 4.5)
		No	8976 (1.1)	812340	

https://doi.org/10.1371/journal.pone.0195827.t002

Among patients evaluated in the ED with a football injury, 6,657 patients presented with skull and/or facial injuries during the 4-year study period (mean 1,665/year); this number had a yearly decline from 1,737 in 2010 to 1,497 in 2013. Similarly, among patients requiring admission to the hospital with diagnosis of skull and/or facial injuries, a declined from 72 in 2010 to 40 in 2013 was seen.

Due to small counts, co-occurring injuries [All Spine with Skull and Face: 10 (0.0%), Concussion with Skull and Face: 358 (0.04%), Concussion with All Spine: 134 (0.02%), Concussion with Spinal Cord: 92 (0.01%)] are not tabulated.

[Reference Table 2 for injury specific features of admission likelihood]

The number of patients with football injuries requiring cranial and spinal surgery over the course of 4 years was relatively low, at 54 and 155, respectively. Direct injuries related to football trauma accounted for a total of 10 deaths during this time period.

Discussion

In this study, we found that American tackle football-related injuries accounted for 0.15% of all ED visits, of which 1.13% of the patients required admission to the hospital. 80% of the ED visits are from the pediatric population. Diagnosis of spinal cord injury, private insurance status, presentation to trauma center or teaching institution, and location in the southern or western regions of the country increased the likelihood of inpatient admission.

American Football remains America's favorite sport [1] despite recent declines in participation. In 2014, there were 2.13 million youth (≤14 years old) participants in tackle football, compared to 3 million in 2010 [14]while high school level participation has demonstrated a more modest decline with 1.11 million athletes in 2014 compared to 1.13 million in 2010 [15]. Notwithstanding, an estimated average 3.75 million individuals participated in organized tackle football each year between 2010 and 2013 [16].

In our study, we found an estimated 819,000 patients requiring visitation to the emergency department and 9,216 requiring admission over the course of four years. Given an estimated denominator of 15 million participants during this same time period, the risk of sustaining a football injury necessitating emergency room visitation and inpatient admission may be predicted at 5.5% and 0.076% per year, respectively. Fortunately, more severe injuries requiring inpatient admission are relatively very low. Unsurprisingly, the largest proportion of ED visits involved pediatric patients, reflecting this population as the majority demographic in terms of participation in tackle football.

Possibly relevant to at least some of the 68,830 concussions reported in Table 2, recent concern regarding the dangers of American tackle football has led to significant interest in epidemiological and biomechanical studies of concussion and understanding the consequences of repetitive sub-concussive impacts [17–26]. In a 2016 study from the University of Florida, more than one out of every four players sustained a concussion over the course of 9 years[17]. At the NFL level, the incidence of concussions has been reported as 0.41 concussions/game [20] A review by Forbes and colleagues (2012) concluded that 0.2% of all collisions on the football field result in concussion [22], an especially sobering finding considering that high school lineman sustain an average of over 850 impacts per season [27]. Biomechanical data, specifically with helmet accelerometers, have recently been used to collect information on the severity of head impacts; however, this data has been unreliable in predicting whether an athlete suffers a concussion [22]. Indeed, in one study, the range of impact severity for high school players sustaining a concussion varied between 48*g* to 146*g* of translational acceleration, underscoring the concept that multiple factors are involved in determining whether or not an athlete experiences a concussion [26].

During the study period included in our analysis, an average of 17,207 patients per year presented to an ED with a football-related concussion, representing 68,830 of 819,000 (8.4%) of all football related presentations.

In our study, we did see a yearly increase in the number of patients with concussions presenting to the ED. One can only speculate as to whether this was because of an actual increase in the number of concussions sustained or because of increased vigilance regarding head injuries in those playing football. Post concussive return to play laws have greatly improved care of these patients by ensuring a physician clears the patient prior to return, but does not mandate early provider post-concussion evaluation or participation in the diagnostic process. This is indeed an improvement from protocols in the 1990's where players potentially could return to the field on the following play series, placing the player at higher chance for second impact syndrome [28]. A single concussion can increase suicide risk by three times, independent of past psychiatric history[20]. This information may further involve physicians in early treatment of concussion and necessitates documentation of concussion when obtaining a patient's medical history.

Amongst the patients presenting to EDs with neurologic injuries, the diagnosis associated with the highest rate of admission (43%) was SCI. Fortunately, the overall incidence of catastrophic SCI secondary to tackle football injuries has dropped precipitously since the 1970's, likely due to rule changes limiting the use of the head as initial contact point during tackling [29]. The rate of catastrophic injury has been estimated between 0.5–1.1 per 100,000 in high school and 1.5–4.72 per 100,000 in college [30].

An average of 383 patients per year were diagnosed with non-concussion intracranial injury, indicating more severe acute injury, which includes intracranial hemorrhage or cerebral edema. Over our 4-year study period, 54 patients had an intracranial injury requiring an operation, representing only 0.007% of all patients who presented to EDs for evaluation of football-related injuries, and 0.08% of those who presented with a diagnosis of intracranial injury; 155 patients had spinal injury requiring operative intervention, representing 0.02% of all football injured patients presenting to the ED and 8.1% of patients who presented with a diagnosis of spinal injury. Assuming 3.75 million participants per year, the rate of neurologic injury requiring any operative intervention was inferred to be 0.0014%, (209/15M) over the four-year study period. Overall, these data indicate that acute injuries relating to the neuroaxis secondary to tackle football are very unlikely to warrant surgery; however, there is no data regarding the long-term effects of tackle football and its potential impact on the development of degenerative pathology eventually requiring surgery. This particular concept is further underscored by data showing that players competing in more than 5 NFL seasons were almost 2.5 times greater to have whole-person impairment. The most common symptomatic joints were lumbar and cervical spine [31].

Teaching institutions and trauma centers were both more likely to admit patients. Presumably, patients with injury severity are taken to both of these types of centers so this information was not unexpected. Interestingly, the region of the country impacted likelihood of admission, with hospitals in the South and West demonstrating a significantly higher likelihood of ED visits turning into admissions than the Northeast or Midwest. Whether this is due to differing practice pattern, injury management, or differing severity of injuries is out of the scope of this investigation, but may be the source of future study.

The hospital charges from both ED visits and admissions over the 4-year study period totaled almost $1.7 billion, amounting to $90 dollars/participant per year. However, though rare, the very few patients with devastating TBI or SCI undoubtedly pose a significant financial burden on our healthcare system. The financial ramifications associated with these particular patients are important to note, given the extended, often life-long, healthcare and

rehabilitation costs associated with their care. Although total charges are included in the NEDS database and are reported here, the necessary cost to charge ratios were not included, preventing us from reporting ED costs.

The National Center for Catastrophic Sports Injury Research tracks fatalities relating to American tackle football. From 1990–2010, there were 243 football related fatalities [30]; 67% were from indirect causes, such as heat exhaustion, cardiac arrhythmias or dehydration, while 33% were from direct traumatic football injuries such as TBI, spinal trauma, or internal bleeding. From 1945–1984, 643 deaths were attributed directly to football injuries at a rate of 16.5 deaths per year [28]. Using the NEDS and NIS databases, we identified 10 football-related deaths from 2010–2013, indicating about 2.5 deaths per year. However, specific causes for each of these cases in our study population were not defined. Though the likelihood of death has decreased in modern American tackle football, we are only beginning to grasp the long-term impact of sustaining non-devastating and acutely nonclinical neurological injuries such as repetitive minor head trauma.

The strengths of this study are that the NEDS and NIS databases are large, well-documented and, by design, comprise a 20% random sample of the community hospital population, making national estimates possible. Limitations include that these data are restricted to diagnostic and procedure codes and that individual patients, being de-identified, cannot be tracked over time or across databases from the NEDS to NIS files, the relatively recent introduction of the tackle-football E-code, possibly missing E-codes, possible misclassifications and unidentifiable repeated visits, and care sought outside of the ED or community hospital system are not included.

Conclusion

Despite recent concern over neurological injuries related to American tackle football, the number of admissions to the hospital remained relatively low over our study period. Operative intervention on more severe neurological injury proved to be extremely infrequent. Most of the ED visits and hospital admission were from the pediatric age group. Acute, devastating neurological injuries are infrequent in American tackle football, but continued investigation in the long-term health impact of football related concussion and other repetitive lower impact trauma is warranted. To that end, further analysis of trends in ED evaluations, admissions, and operations as well as tracking the financial burden of football-related injuries on the U.S. healthcare system has significant merit.

Author Contributions

Conceptualization: Michael J. McGinity, Ali Seifi.

Data curation: Joel E. Michalek.

Formal analysis: Joel E. Michalek, Aron M. Trevino.

Investigation: Michael J. McGinity, Ali Seifi.

Methodology: Ali Seifi.

Project administration: Ali Seifi.

Resources: Ali Seifi.

Software: Joel E. Michalek.

Supervision: Ali Seifi.

Validation: Michael J. McGinity, Ali Seifi.

Visualization: Ali Seifi.

Writing – original draft: Michael J. McGinity, Ali Seifi.

Writing – review & editing: Ramesh Grandhi, Jesse S. Rodriguez, Ashley C. McGinity, Ali Seifi.

References

1. Shannon-Missal, L. Pro Football is Still America's Favorite Sport [Internet]. 2016 Jan. Report No.: 7. http://www.theharrispoll.com/sports/Americas_Fav_Sport_2016.html
2. Clinicopathological Evaluation of Chronic Traumatic Encephalopathy in Players of American Football. JAMA. 2017 Jul 25; 318(4):360–370. PMID: 28742910
3. Darrow CJ, Collins CL, Yard EE, Comstock RD. Epidemiology of severe injuries among United States high school athletes: 2005–2007. Am J Sports Med. 2009 Sep; 37(9):1798–805. https://doi.org/10.1177/0363546509333015 PMID: 19531659
4. Lawrence DW, Hutchison MG, Comper P. Descriptive Epidemiology of Musculoskeletal Injuries and Concussions in the National Football League, 2012–2014. Orthop J Sports Med [Internet]. 2015 May 5 [cited 2016 Jun 28]; 3(5). Available from: http://ojs.sagepub.com/lookup/doi/10.1177/2325967115583653
5. King H, Campbell S, Herzog M, Popoli D, Reisner A, Polikandriotis J. Epidemiology of Injuries in High School Football: Does School Size Matter? J Phys Act Health. 2015 Aug; 12(8):1162–7. https://doi.org/10.1123/jpah.2014-0356 PMID: 25329725
6. Dick R, Ferrara MS, Agel J, Courson R, Marshall SW, Hanley MJ, et al. Descriptive epidemiology of collegiate men's football injuries: National Collegiate Athletic Association Injury Surveillance System, 1988–1989 through 2003–2004. J Athl Train. 2007 Jun; 42(2):221–33. PMID: 17710170
7. Feeley BT, Kennelly S, Barnes RP, Muller MS, Kelly BT, Rodeo SA, et al. Epidemiology of National Football League training camp injuries from 1998 to 2007. Am J Sports Med. 2008 Aug; 36(8):1597–603. https://doi.org/10.1177/0363546508316021 PMID: 18443276
8. Lincoln AE, Caswell SV, Almquist JL, Dunn RE, Norris JB, Hinton RY. Trends in concussion incidence in high school sports: a prospective 11-year study. Am J Sports Med. 2011 May; 39(5):958–63. https://doi.org/10.1177/0363546510392326 PMID: 21278427
9. Powell JW, Barber-Foss KD. Traumatic brain injury in high school athletes. JAMA. 1999 Sep 8; 282 (10):958–63. PMID: 10485681
10. McKee AC, Cantu RC, Nowinski CJ, Hedley-Whyte ET, Gavett BE, Budson AE, et al. Chronic traumatic encephalopathy in athletes: progressive tauopathy after repetitive head injury. J Neuropathol Exp Neurol. 2009 Jul; 68(7):709–35. https://doi.org/10.1097/NEN.0b013e3181a9d503 PMID: 19535999
11. Stern RA, Riley DO, Daneshvar DH, Nowinski CJ, Cantu RC, McKee AC. Long-term consequences of repetitive brain trauma: chronic traumatic encephalopathy. PM R. 2011 Oct; 3(10 Suppl 2):S460–467. https://doi.org/10.1016/j.pmrj.2011.08.008 PMID: 22035690
12. National Inpatient Sample (NIS). Healthcare Cost and Utilization Project (HCUP). 2012. Agency for Healthcare Research and Quality, Rockville, MD. [Internet]. https://www.hcup-us.ahrq.gov/nisoverview.jsp
13. Nationwide Emergency Department Sample (NEDS). Healthcare Cost and Utilization Project (HCUP). 2007, 2008, 2009. Agency for Healthcare Research and Quality, Rockville, MD. [Internet]. https://www.hcup-us.ahrq.gov/nedsoverview.jsp
14. The Physical Activity Council's annual study tracking sports, fitness, and recreation participation in the US [Internet]. Physical Activity Council; 2016. (Participation Report). http://www.physicalactivitycouncil.com/PDFs/current.pdf
15. High School Athletics Participation Survey Results [Internet]. 2014 2015. (National Federation of State High School Associations). http://www.nfhs.org/ParticipationStatistics/

16. National Collegiate Athletic Association. Student-athlete participation 1981–1982–2012–13: NCAA sports sponsorship and participation rates report. [Internet]. 2014. http://www.ncaapublications.com/productdownloads/PR2014.pdf

17. Houck Z, Asken B, Bauer R, Pothast J, Michaudet C, Clugston J. Epidemiology of Sport-Related Concussion in an NCAA Division I Football Bowl Subdivision Sample. Am J Sports Med. 2016 May 3;

18. Kerr ZY, Zuckerman SL, Wasserman EB, Covassin T, Djoko A, Dompier TP. Concussion Symptoms and Return to Play Time in Youth, High School, and College American Football Athletes. JAMA Pediatr. 2016 May 2;

19. Lehman EJ, Hein MJ, Gersic CM. Suicide Mortality Among Retired National Football League Players Who Played 5 or More Seasons. Am J Sports Med. 2016 May 5;

20. Fralick M, Thiruchelvam D, Tien HC, Redelmeier DA. Risk of suicide after a concussion. CMAJ Can Med Assoc J J Assoc Medicale Can. 2016 Apr 19; 188(7):497–504.

21. Pellman EJ, Powell JW, Viano DC, Casson IR, Tucker AM, Feuer H, et al. Concussion in professional football: epidemiological features of game injuries and review of the literature—part 3. Neurosurgery. 2004 Jan; 54(1):81–94; discussion 94–96. PMID: 14683544

22. Forbes JA, Awad AJ, Zuckerman S, Carr K, Cheng JS. Association between biomechanical parameters and concussion in helmeted collisions in American football: a review of the literature. Neurosurg Focus. 2012 Dec; 33(6):E10: 1–6. https://doi.org/10.3171/2012.9.FOCUS12288 PMID: 23199422

23. Collins CL, McKenzie LB, Ferketich AK, Andridge R, Xiang H, Comstock RD. Concussion Characteristics in High School Football by Helmet Age/Recondition Status, Manufacturer, and Model: 2008–2009 Through 2012–2013 Academic Years in the United States. Am J Sports Med. 2016 Jun; 44(6):1382–90. https://doi.org/10.1177/0363546516629626 PMID: 26905506

24. Johnson LSM. Return to play guidelines cannot solve the football-related concussion problem. J Sch Health. 2012 Apr; 82(4):180–5. https://doi.org/10.1111/j.1746-1561.2011.00684.x PMID: 22385091

25. Bentz JE. Concussion in American Football and Sports. J Lanc Gen Hosp. 2013; 8(3).

26. Broglio SP, Eckner JT, Surma T, Kutcher JS. Post-concussion cognitive declines and symptomatology are not related to concussion biomechanics in high school football players. J Neurotrauma. 2011 Oct; 28(10):2061–8. https://doi.org/10.1089/neu.2011.1905 PMID: 21644811

27. Broglio SP, Eckner JT, Martini D, Sosnoff JJ, Kutcher JS, Randolph C. Cumulative head impact burden in high school football. J Neurotrauma. 2011 Oct; 28(10):2069–78. https://doi.org/10.1089/neu.2011.1825 PMID: 21787201

28. Saal JA. Common American football injuries. Sports Med Auckl NZ. 1991 Aug; 12(2):132–47.

29. Rihn JA, Anderson DT, Lamb K, Deluca PF, Bata A, Marchetto PA, et al. Cervical spine injuries in American football. Sports Med Auckl NZ. 2009; 39(9):697–708.

30. Boden BP, Breit I, Beachler JA, Williams A, Mueller FO. Fatalities in high school and college football players. Am J Sports Med. 2013 May; 41(5):1108–16. https://doi.org/10.1177/0363546513478572 PMID: 23477766

31. Domb BG, Carter C, Finch NA, Hammarstedt JE, Dunne KF, Stake CE. Whole-Person Impairment in Younger Retired NFL Players: The Orthopaedic Toll of a Professional Football Career. Orthop J Sports Med. 2014 May; 2(5):2325967114534824.
